# Optic Nerve Magnetisation Transfer Ratio after Acute Optic Neuritis Predicts Axonal and Visual Outcomes

**DOI:** 10.1371/journal.pone.0052291

**Published:** 2012-12-18

**Authors:** Yejun Wang, Anneke van der Walt, Mark Paine, Alexander Klistorner, Helmut Butzkueven, Gary F. Egan, Trevor J. Kilpatrick, Scott C. Kolbe

**Affiliations:** 1 The Centre for Neuroscience Research, University of Melbourne, Parkville, Victoria, Australia; 2 Department of Medicine, University of Melbourne, Parkville, Victoria, Australia; 3 The Department of Neurology, The Royal Melbourne Hospital, Parkville, Victoria, Australia; 4 The Royal Victoria Eye and Ear Hospital, East Melbourne, Victoria, Australia; 5 The Sydney Eye Hospital, Sydney, New South Wales, Australia; 6 Monash Biomedical Imaging, Monash University, Clayton, Victoria, Australia; Institute Biomedical Research August Pi Sunyer (IDIBAPS) - Hospital Clinic of Barcelona, Spain

## Abstract

Magnetisation transfer ratio (MTR) can reveal the degree of proton exchange between free water and macromolecules and was suggested to be pathological informative. We aimed to investigate changes in optic nerve MTR over 12 months following acute optic neuritis (ON) and to determine whether MTR measurements can predict clinical and paraclinical outcomes at 6 and 12 months. Thirty-seven patients with acute ON were studied within 2 weeks of presentation and at 1, 3, 6 and 12 months. Assessments included optic nerve MTR, retinal nerve fibre layer (RNFL) thickness, multifocal visual evoked potential (mfVEP) amplitude and latency and high (100%) and low (2.5%) contrast letter acuity. Eleven healthy controls were scanned twice four weeks apart for comparison with patients. Patient unaffected optic nerve MTR did not significantly differ from controls at any time-point. Compared to the unaffected nerve, affected optic nerve MTR was significantly reduced at 3 months (mean percentage interocular difference = −9.24%, *p* = 0.01), 6 months (mean = −12.48%, *p*<0.0001) and 12 months (mean = −7.61%, *p* = 0.003). Greater reduction in MTR at 3 months in patients was associated with subsequent loss of high contrast letter acuity at 6 (ρ = 0.60, *p* = 0.0003) and 12 (ρ = 0.44, *p* = 0.009) months, low contrast letter acuity at 6 (ρ = 0.35, *p* = 0.047) months, and RNFL thinning at 12 (ρ = 0.35, *p* = 0.044) months. Stratification of individual patient MTR time courses based on flux over 12 months (stable, putative remyelination and putative degeneration) predicted RNFL thinning at 12 months (*F*_2,32_ = 3.59, *p* = 0.02). In conclusion, these findings indicate that MTR flux after acute ON is predictive of axonal degeneration and visual disability outcomes.

## Introduction

Optic neuritis (ON) is a frequent early presentation of multiple sclerosis (MS) with measureable clinical signs that allow for early diagnosis [1]. Although patients with ON often have a good visual acuity outcome, more sensitive measures of visual dysfunction such as visual evoked potentials (VEP) and contrast sensitivity commonly reveal persistent abnormalities [1]–[3]. Such persistent abnormalities are likely attributable to optic nerve demyelination and axonal degeneration [4].

Optic nerve axonal degeneration and demyelination can be assessed after acute ON using paraclinical tools such as optical coherence tomography (OCT) or VEP [4]–[10]. Retinal nerve fibre layer (RNFL) thinning, which is a surrogate marker for optic nerve axonal degeneration, can be measured and tracked using OCT [4]–[6], and can predict visual recovery after ON [5], [6]. Visual evoked potential amplitude loss and latency prolongation are believed to reflect axonal loss and demyelination of the optic nerve respectively in ON patients [8], [9]. Acutely however, retinal nerve fibre layer (RNFL) thickness measurements are confounded by inflammatory oedema and VEP can be completely extinguished due to acute demyelination and associated conduction block. Hence, there is a need for earlier predictors of poor visual outcomes and axonal degeneration.

Magnetic resonance imaging techniques such as magnetisation transfer ratio (MTR) imaging can reveal the degree of proton exchange between free water and macromolecules such as lipids [11]. Given that myelin and axons provide numerous lipid membranes, MTR can provide information regarding the degree of optic nerve demyelination and axonal loss that are relevant to visual and paraclinical outcomes after optic neuritis [12], [13]. However, the specific relationships between flux in optic nerve MTR after acute ON and visual and paraclinical outcomes have not been clearly established [12]–[16]. In particular, the results of a longitudinal study by Hickman and colleagues provided some evidence that MTR might secondarily increase after initially decreasing potentially indicating optic nerve remyelination.

The study by Hickman *et al.* [12] attempted to model the MTR timecourses of all patients using a curve fit representative of secondary remyelination. However, more recent evidence based on multifocal VEP data [8] suggests that patients exhibit variable recovery patterns after acute ON with some patients exhibiting decreasing latency indicative of remyelination and some patients exhibiting persistent amplitude loss indicative of irreversible axonal degeneration. In this study, we therefore aimed to investigate flux in MTR over 12 months following acute ON to examine the determinants of visual and paraclinical outcomes. We hypothesised that patients with early MTR reduction or MTR timecourses indicative of ongoing degeneration would exhibit greater visual disability and axonal degeneration outcomes at 6 and 12 months after acute ON.

**Table 1:** Baseline demographics of Acute ON patients who completed 12 months of follow-up.

Characteristic	N = 37
Age (years) (mean, SD)	35 (9)
Female (N,%)	26 (70)
Symptom duration prior to baseline testing (median days, range)	5 (1–14)
Methylprednisolone treatment (N,%)	28 (76)
Diagnosis at presentation	
Clinically isolated syndrome (N,%)	31 (84)
Early RRMS* (N,%)	6 (16)
Conversion to Clinically Definite MS at 12 months (N,%)	13 (42)
Baseline visual acuity	
LogMAR Visual acuity derived from Sloan 100% contrast letter acuity chart (mean, 95% CI, IQR range)	0.61 (0.12, −0.2–1.2)
2.5% Low contrast letter acuity** (mean number of letters correct out of 60, 95% CI, IQR range)	4.2 (3.1, 0 to 0)

* Patients with RRMS with disease duration less than 2 years.

** Sloan charts 2.5% contrast.

**Table 2:** Summary statistics for optic nerve MTR in patients. MTR values are percent units.

Time-point	Unaffected Optic Nerve MTR(Mean ±95% CI, difference from control) (%)	Affected Optic Nerve MTR(Mean ±95% CI, difference from unaffected) (%)	Interocular Asymmetry (Mean±95% CI)
Baseline	43.4±2.3, n.s.	43.2±2.4, n.s	0.60±5.60%
1 month	44.8±2.6, n.s.	42.8±2.5, n.s	−2.55±7.20%
3 month	44.9±2.5, n.s	41.2±3.3, p = 0.01	−9.24±5.93%
6 month	43.3±2.2, n.s	37.7±2.7, p<0.0001	−12.48±5.27%
12 month	42.5±2.4, n.s	38.7±2.4, p = 0.003	−7.61±5.75%

n.s. = not significant.

## Methods

### Ethics Statement

This study was conducted in accordance with Declaration of Helsinki and was approved by the human research ethic committees of the Royal Victorian Eye and Ear (recruitment and testing site) and Royal Melbourne (clinical management site) Hospitals and the Murdoch Children’s Research Institute (MRI scanning site). All study participants provided voluntary, written consent.

### Subjects and Study Protocol

Forty adults presenting with a first episode of unilateral acute ON were recruited within 2 weeks of symptom onset. Patients with prior ophthalmological or neurological conditions or who developed recurrent ON during the study were excluded. Patients with a high risk of developing MS according to the O’Riordan criteria (at lease 2 hyperintense lesions on MRI) [17] or with early relapsing remitting MS (disease duration of less than 2 years) according to the McDonald Criteria were included [18]. Two patients withdrew from the study after 1 month and a third developed recurrent ON resulting in exclusion. Atypical presentations were confirmed by optic nerve gadolinium enhancement or abnormal multifocal visual evoked potentials (mfVEP). Participants were tested at baseline, 1, 3, 6 and 12 months. Eleven healthy volunteers were recruited and tested twice, four weeks apart to assess inter- and intra -subject variability.

### Clinical Assessment

Best corrected VA was performed in the same well-lit room at each visit using high contrast (Sloan 100%) and low contrast (Sloan 2.5%) letter acuity (LCLA) charts [19], [20]. Snellen VA equivalents (converted to LogMAR scale) were derived from high contrast VA. In the LCLA tests the numbers of letters correctly recognised (maximum 60/chart) were recorded for each eye. Correctly identified Ishihara plates (out of 38) were used to assess colour vision.

**Figure 1:**
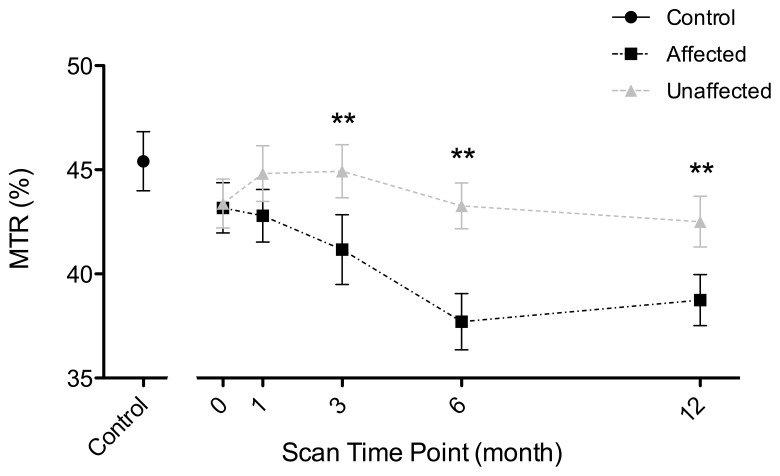
Serial measurements of MTR in patient affected and unaffected optic nerves during the 12 months following acute optic neuritis. Significant differences were observed at three, six and 12 months (** indicates p<0.01 for paired t-test). The control distribution is indicated on the left for comparison. Errorbars indicate one standard error of the mean (SEM).

**Table 3:** Spearman rank correlations between MTR asymmetry at 3,6, and 12 months and visual, OCT and mfVEP outcomes at 6 and 12 months.

MTR		6 months					12 months				
		Sloan 100%	Sloan 2.5%	RNFL Thickness	mfVEP amplitude	mfVEP latency	Sloan 100%	Sloan 2.5%	RNFL Thickness	mfVEP amplitude	mfVEP latency
3 months	ρ	**0.60**	**0.35**	0.31	0.29	−0.22	**0.44**	0.30	**0.35**	0.28	−0.29
	*p*	**0.0003**	**0.047**	0.08	0.12	0.33	**0.009**	0.09	**0.044**	0.12	0.14
	n	**32**	**32**	32	30	22	**34**	34	**34**	33	28
6 months	ρ	−0.02	−0.19	−0.01	0.06	**−0.48**	−0.14	−0.04	−0.01	−0.08	−0.31
	*p*	0.94	0.28	0.96	0.74	**0.029**	0.45	0.82	0.96	0.65	0.13
	n	33	33	33	30	**21**	33	33	33	32	26
12 months	ρ						0.07	0.09	**0.41**	0.31	−0.36
	*p*						0.68	0.61	**0.014**	0.08	0.06
	n						35	35	**35**	34	28

### Optical Coherence Tomography (OCT)

RNFL thickness measurement was performed on an OCT-3 scanner (Stratus™, software version 3.0, Carl ZeissMeditec Inc., Dublin, CA) with a fast RNFL protocol consisting of three circular scans centred on the optic disc with 3.4 mm diameters. Signal strength of 7 or more was accepted.

### Multi-focal Visual Evoked Potential (mfVEP)

All mfVEPs were recorded using the Accumap ™ (ObjectVision, Sydney, Australia) with procedures as previously described by Fraser and co-workers [21]. Four gold cup electrodes were placed in a cross formation around the inion to simultaneously collected VEPs from 58 sectors of the visual field. The central area of 1**°** was only used as a fixation monitor. Recordings of 54s with 10–12 runs for each eye were collected to reach a defined signal to noise ratio. VEPs were calculated for each sector of the visual field and for the whole eye using the OPERA program (ObjectVision, Sydney, Australia). Segments with amplitude signal less than 1.96 times the standard deviation (SD) of the trace within the interval 400–1000 ms were considered as non-recordable (baseline [n = 20], 1 month [n = 5], 3 months [n = 7], 6 months [n = 4], 12 months [n = 1]) and excluded from further data analysis.

### Magnetic Resonance Imaging (MRI)

MRI was performed using a Siemens 3T MRI system with a 32-channel transmit-receive head coil. MTR was calculated from two coronally acquired 2D gradient echo (GE) scans, one with and one without a pre-saturation pulse to excite macromolecular protons. Imaging parameters were: TR = 710 ms, TE = 8.4 ms, NEX = 1, matrix size = 256×192, field of view = 180×135 mm^2^, in-plane voxel dimensions = 0.7×0.7 mm^2^, slice thickness = 3 mm (interleaved contiguous with whole brain coverage), acquisition time = 4 mins 1 sec for each scan. MTR was calculated by using the formula: 100×[(M_0_-M_s_)/M_0_] percentage units (%), where M_s_ and M_0_ represent signal intensities from images with and without pre-saturation pulse respectively. The mean MTR of each optic nerve was calculated from the MTR within a manually delineated region of interest (ROI). Regions of interest four voxels in size were delineated from the centre of the optic nerves in each slice posterior to the globe and anterior to the optic canal. Twenty randomly selected patients optic nerves were rated twice by a single observer (YW), and by an independent observer (SK) 3 months apart to calculate intra- and inter-rater reliability. Some patients did not complete MTR scanning at each time point (baseline [n = 3], 1 month [n = 5], 3 month [n = 3], 6 month [n = 4], 12 month [n = 2]).

**Figure 2:**
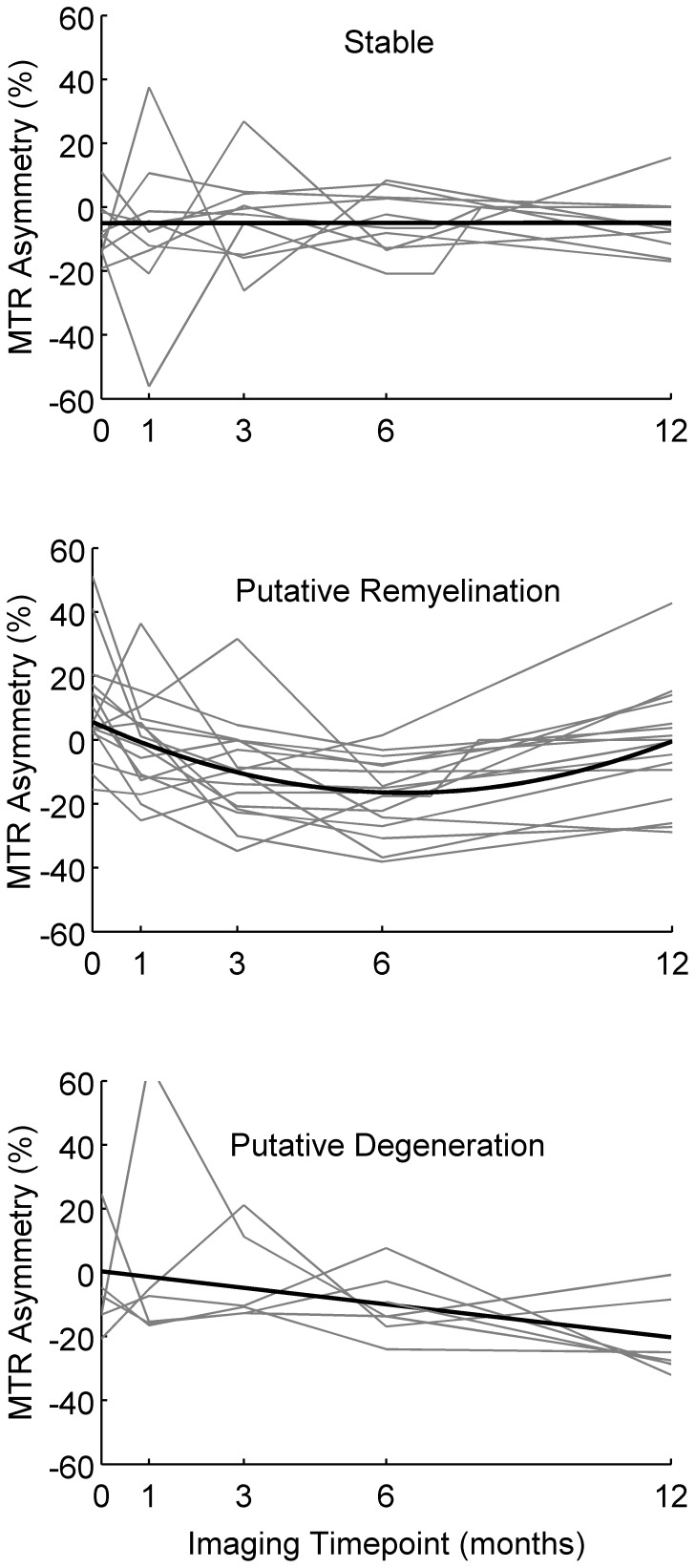
Individual patient MTR asymmetry time courses (grey lines) and mean fitted time courses (black lines) for three subgroups defined based on the pattern of MTR flux. *p<0.05.

**Figure 3:**
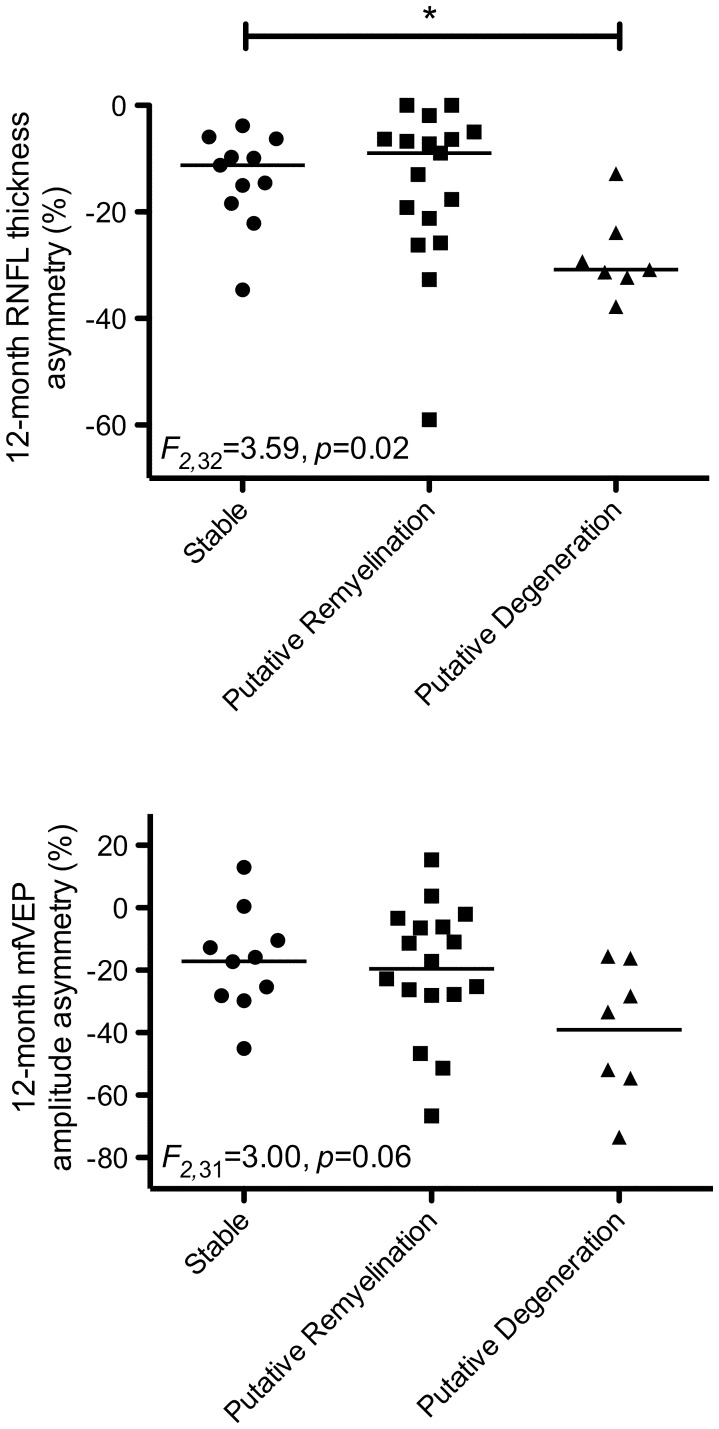
Scatterplots showing distributions for RNFL thinning (upper) and mfVEP amplitude loss (lower) based on subgroups. *p<0.05.

### Statistical Analyses

#### Reproducibility analyses

Inter- and intra-rater reliability was assessed using the Bland-Altman limits of agreement method [22]. The Kolmogorov-Smirnov test was used to test for normality of the MTR data distributions. Mean and 95% confidence intervals (CI) for control MTR were calculated using a bootstrapping method [23] with 5000 samples containing one randomly chosen optic nerve from each control subject.

#### Group comparisons

Unpaired t-tests were used to compare unaffected nerve MTR at each time-point to the control MTR. Mixed-effects two-way analysis of variance (ANOVA) was used to test for time-point and optic nerve clinical status interaction. Post-hoc paired t-tests were used to compare affected and unaffected optic nerve MTR within and between time points. To minimise inter-subject variability not related to disease, normalised asymmetry coefficients were used for all outcome measures: [(affected-unaffected)/unaffected]*100 (%) [9].

#### Correlation analyses

Non-parametric Spearman’s tests were used to test correlations between MTR at 3, 6 and 12 months and OCT, mfVEP and VA measures at 6 and 12 months.

#### Subgroup analysis based on MTR flux

In order to assess the putative effects of remyelination on clinical and paraclinical outcomes, patients were grouped into three subtypes based on the longitudinal pattern of MTR asymmetry over 12 months: (1) stable, (2) putative remyelination, and (3) putative degeneration. The method for grouping was as follows. (1) Stable: MTR flux over 12 months was linear and reducing at a rate of less than 1% per month. (2) Putative remyelination: MTR flux was better fit (R^2^ increase by >0.1) by a quadratic function. (3) Putative degeneration: MTR flux was linear and reducing at a rate of >1% per month. One-way ANOVA was used to compare clinical and paraclinical variables at 12 months based on the subgroups defined above.

All statistical analyses were performed using IBM SPSS Statistics 20.

## Results

### Patient Demographics (Table 1)

Thirty-seven patients completed 12 months of follow-up. Thirteen CIS patients (42%) converted to clinically definite MS during the 12 months follow-up period and 38% (n = 14) had commenced immunomodulatory treatment by 12 months. The median EDSS at 12 months was 0 (interquartile range 0 to 1). Baseline LogMAR VA was 0.61 (Snellen 6/24) (95% confidence interval (CI) 0.25 to 1.0)) and improved to −0.1 (Snellen 6/5) (95% CI −0.2 to 0.0) at 12 months. Poor LCLA recovery was seen in 37% (n = 13) at 6 months and 32% (n = 12) at 12 months.

### MTR Reliability Analysis

The coefficient of variation for intra-rater reliability was 6.0%, limits of agreement 2.6% to 9.4%. The coefficient of variation for inter-rater reliability was 11.0%, limits of agreement 3.9% to 18.1%.

### Control MTR and Comparisons with Patients

MTR values were normally distributed. For the control group, paired t-tests revealed no significant inter-ocular or inter-scan differences in MTR. Bootstrapped mean (95% CI) for control MTR was 45.4% (42.6% to 58.2%). No differences between the unaffected nerve MTR at any time point and control values were found (Table 2).

### Patient Optic Nerve MTR Flux Over 12 Months (Figure 1)

A significant interaction between time-point and optic nerve clinical status (*F*_9,335_ = 3.404, *p* = 0.001) was found. Affected nerve MTR was significantly reduced compared to unaffected at 3 months (mean percentage interocular difference [95% CI] = −9% [−15% to −3%], *p* = 0.01), 6 months (mean [CI] = −12% [−18% to 7%], *p*<0.0001) and 12 months (mean [CI] = −8% [13% to 2%], *p* = 0.003) (Table 2). Mixed-effects one-way ANOVA revealed a significant change in affected nerve MTR over 12 months (*F_4,62_* = 3.156, *p* = 0.016). Unaffected nerve MTR did not change over 12 months (*F_4,162_* = 0.800, *p* = 0.527).

### Correlations between Affected Optic Nerve MTR, Clinical and Paraclinical Outcomes (Table 3)

Given that inter-ocular MTR was not altered at baseline or 1 month, correlation analyses were only performed for later time points. At 3 months, reduced MTR was associated with poorer high contrast VA at 6 months (ρ = 0.60, *p* = 0.0003) and 12 months (ρ = 0.44, *p* = 0.009), poorer LCLA at 6 months (ρ = 0.35, *p* = 0.047) and RNFL thinning at 12 months (ρ = 0.35, *p* = 0.04). At 6 months reduction in MTR was associated with concurrent mfVEP latency prolongation (ρ = −0.48, *p* = 0.03) but was no longer predictive of visual outcome. At 12 months reduced MTR was associated with concurrent RNFL thinning (ρ = 0.41, *p* = 0.014) but not visual outcome.

### Subgroup Analysis Based on MTR Flux

Based on patterns of MTR flux, patients were divided into three subgroups: stable (n = 11, mean fit: *f(t)* = 0.01*t* −4.7), putative remyelination (n = 18, mean fit: *f(t)* = 0.53*x^2^*–6.8*t* +5.6, nadir at 195 days), putative degeneration (n = 7, mean fit: *f(t)* = −1.7*t* +0.38). Individual patient MTR asymmetry time-courses and average fits for each group are illustrated in figure 2. One-way ANOVA reported a significant effect of subgroup on 12-month RNFL thickness asymmetry (*F_2,_*_32_ = 3.59, *p* = 0.02). Post-hoc tests revealed significantly more RNFL thinning in the degenerative subgroup compared to the stable subgroup (*p*<0.05) (Figure 3). A near-significant trend was also observed for mfVEP amplitude attenuation (*F_2,_*_31_ = 3.00, *p* = 0.06) (Figure 3). High and low contrast visual acuity and mfVEP latency at 12 months did not differ between subgroups.

## Discussion

This study aimed to assess MTR alterations in the 12 months following acute ON and determine whether early flux in MTR predicts subsequent visual and paraclinical outcomes. At a group level, MTR in affected optic nerves remained unchanged compared to unaffected nerves until 3 months after acute ON, by which time a significant decrease in MTR was detected, which further decreased by 6 months before stabilising between 6 and 12 months. We observed significant correlations between reduction in affected nerve MTR at 3 months and loss of high contrast letter acuity at 6 and 12 months, low contrast letter acuity at 6 months and RNFL thinning at 12 months. Taken together, these data suggest that MTR measurements at 3 months could be informative of processes contributing to axonal degeneration and functional disability after ON. Indeed, at 12 months persistent MTR reduction was associated with RNFL thinning.

We subsequently investigated whether optic nerve MTR flux over the entire 12 months was associated with visual and axonal outcomes. We stratified patients into three groups based on the pattern of MTR flux. Ratios of patients in each group were: 30% stable, 50% putative remyelination and 20% putative degeneration. Compared to stable patients, the putative degeneration group displayed significantly greater RNFL thinning and a trend towards greater mfVEP amplitude loss at 12 months. These data therefore suggest that progressive decrease in MTR is indicative of optic nerve axonal degeneration as measured by robust paraclinical markers sensitive to axonal pathology [5], [9], [21]. Although there was no difference between the putative remyelination group and the other two groups, the distribution of RNFL thickness and mfVEP amplitude outcomes in the putative remyelination group more closely resembled the stable group, that may be an indicator of remyelination of intact optic nerve axons. Interestingly, we observed no difference in visual outcomes between the three groups indicating a disparity between the determinants of axonal and visual outcomes as has been reported previously [6].

A previous report by Hickman *et al.* [12] hypothesised that optic nerve MTR might increase post-acutely (between 6 and 12 months) in response to remyelination. The authors used quadratic regression models similar to those used in the current study, but fitted to group averaged optic nerve MTR time course data rather than to individual patient data. The authors found that their models explained the longitudinal profile of MTR flux and argued that the significance of the quadratic model was suggestive of a remyelination effect on MTR. The authors did not stratify patients based on the pattern of MTR flux, as was done here, so it is unclear whether all or only a subset of their patients displayed subsequent normalisation of MTR.

Interestingly, we did not observe any significant MTR decrease prior to 3 months. Previous studies have reported that MTR in acutely affected optic nerves is either not different from [12] or increased [16] compared to unaffected nerves. In brain lesions, MTR has been shown to decrease significantly prior to and increasingly during acute lesion formation. These differences in observations between acute brain and optic nerve lesion MTR indicate differential pathological processes of acute lesion formation in the optic nerves and brain.

MTR measures the proton signal associated with exchange between free water molecules and water molecules bound to macromolecules such as lipid membranes [11]. As such, MTR, while commonly considered to be a marker for myelination, is influenced by a multitude of pathological processes such as inflammation, demyelination, axonal loss and oedema [24]–[26] which are likely to be differentially evident as the disease evolves. Our data suggest that after 3 months, the pathologies contributing to further optic nerve MTR decrease may be too complex for the measure to be informative regarding visual and axonal outcomes. Cross-sectional studies of patients with historical ON have revealed various correlative relationships between optic nerve MTR and visual or paraclinical outcomes. Trip *et al.* [13] observed moderate correlations between MTR reduction and VEP latency prolongation and RNFL thinning leading the authors to conclude that MTR is a composite measure of both myelin and axonal integrity. In contrast, Klistorner and colleagues [15] reported no correlation between MTR and mfVEP latency after removal of patients with overt optic nerve axonal loss suggesting that one of the principal pathological drivers of MTR reduction could be axonal loss.

Magnetisation transfer imaging has several advantages over similar quantitative white matter imaging techniques, such as T1 and T2 mapping [27], making it potentially suitable for clinical optic nerve imaging in the context of ON. First, MT imaging sequences can be performed at high spatial resolution, are routinely available on most current clinical MRI systems and the MTR calculation requires no complex post-processing. Second, MTR can be acquired in short and clinically relevant times, 8 minutes in this study for whole brain coverage. In contrast, other techniques such as myelin water imaging require longer acquisition times. Third, there is clear contrast between optic nerve and surrounding optic nerve sheath and orbital fat enabling clear delineation of the nerve. However, given the current interest in using MTR as a potential marker for remyelination, establishing the pathological substrates of MTR change is an important challenge. Pathological studies in brain tissue suggest that MTR correlates with both myelin [24], [28] and axonal density [26], [28]. Results from this study reflect the complexity of interpreting flux in optic nerve MTR after an acute inflammatory event and highlight the need for further studies aimed at investigating the pathological specificity of MTR during and after acute inflammation in animal models of optic neuritis. In addition, studying optic nerve MTR at time points beyond 12 months could provide valuable information regarding ongoing processes such as remyelination, axonal loss and gliosis.

In conclusion, our results demonstrate the evolution of optic nerve MTR following acute ON and provide evidence that MTR measured 3 months after ON could indicate both visual and axonal outcomes. Further studies are required to evaluate the pathological substrates of optic nerve MTR changes in animal models of ON and determine whether MTR measurements after the initial 12 months are clinically informative.
