# Correction: Optic Nerve Magnetisation Transfer Ratio after Acute Optic Neuritis Predicts Axonal and Visual Outcomes

**DOI:** 10.1371/annotation/1c474016-06ef-4827-8fe8-82c158d7616b

**Published:** 2012-12-28

**Authors:** Yejun Wang, Anneke van der Walt, Mark Paine, Alexander Klistorner, Helmut Butzkueven, Gary F. Egan, Trevor J. Kilpatrick, Scott C. Kolbe

There was an error in the Competing Interests statement. The correct Competing Interests statement is as follows: For this particular study we received small unrestricted grants from Bayer Australia, Biogen Idec, Merck Serono and GlaxoSmithKline. There are no patents, products in development or marketed products to declare. This does not alter our adherence to all the PLOS ONE policies on sharing data and materials, as detailed online in the guide for authors.

